# Gestational Exposure to Particulate Matter 2.5 (PM_2.5_) Leads to Spatial Memory Dysfunction and Neurodevelopmental Impairment in Hippocampus of Mice Offspring

**DOI:** 10.3389/fnins.2018.01000

**Published:** 2019-01-07

**Authors:** Xinrui Zheng, Xia Wang, Tingting Wang, Hongxia Zhang, Hongjuan Wu, Can Zhang, Li Yu, Yingjun Guan

**Affiliations:** ^1^Neurologic Disorders and Regeneration Repair Lab of Shandong Higher Education, Department of Histology and Embryology, Weifang Medical University, Weifang, China; ^2^School of Public Health and Management, Weifang Medical University, Weifang, China; ^3^Genetics and Aging Research Unit, Department of Neurology, Massachusetts General Hospital, Harvard Medical School, Charlestown, MA, United States

**Keywords:** PM_2.5_, hippocampus, neurodevelopment, offspring, inflammation, apoptosis

## Abstract

Prenatal exposure to air pollutants has long-term impact on growth retardation of nervous system development and is related to central nervous system diseases in children. However, it is not well-characterized whether gestational exposure to air pollutants affects the development of nervous system in offspring. Here, we investigated the effects of gestational exposure to particulate matter 2.5 (PM_2.5_) on hippocampus development in mice offspring, through neurobehavioral, ultrastructural, biochemical and molecular investigations. We found that spatial memory in mice offspring from PM_2.5_ high-dosage group was impaired. Next, hippocampal ultrastructure of the mice offspring in puberty exhibited mitochondrial damage related to PM_2.5_ exposure. Interestingly, EdU-positive cells in the subgranular zone (SGZ) of offspring from PM_2.5_ high-dosage group decreased, with NeuN^+^/EdU^+^cells reduced significantly. Furthermore, the numbers of NeuN^+^/TUNEL^+^, GFAP^+^/TUNEL^+^, and Iba1^+^/TUNEL^+^ double-labeled cells increased with PM_2.5_ exposure in a dosage-dependent manner. In addition, gestational exposure to PM_2.5_ resulted in increased levels of both mRNAs and proteins involved in apoptosis, including caspase-3, -8, -9, p53, and c-Fos, and decreased Bcl-2/Bax ratios in the hippocampus of mice offspring. Moreover, gestational exposure to PM_2.5_ was dosage-dependently associated with the increased secretions of inflammatory proteins, including NF-κB, TNF-α, and IL-1β. Collectively, our results suggest that gestational exposure to PM_2.5_ leads to spatial memory dysfunction and neurodevelopmental impairment by exerting effects on apoptotic and neuroinflammatory events, as well as the neurogenesis in hippocampus of mice offspring.

## Introduction

Air pollutants are complex mixtures containing particles, gasses, adsorbed metals and organic pollutants. PM_2.5_ (aerodynamic diameters equal to or less than 2.5 μm) acts as a major type of air pollutants and can absorb large quantities of heavy metals and toxic organic pollutants (Chao et al., 2017). Environmental exposure to PM_2.5_ results in about 42 million deaths, accounting for approximately 7.6% of the total number of deaths worldwide (Cohen et al., 2017). Air pollution exerts different effects on people of different ages, with children, pregnant women and the elderly as the most susceptible populations (Allen et al., 2013; Kalkbrenner et al., 2015; Ostro et al., 2015). It has been increasingly recognized that CNS can be the target of PM_2.5_, supported by epidemiological studies suggesting an association between PM_2.5_ exposure and CNS-related disorders including cognitive impairment, stroke, as well as some mental disorders such as schizophrenia, depression and autism (Babadjouni et al., 2017; Sram et al., 2017). The developing brain undergoes precise regulations of cellular events such as proliferation, differentiation, migration and maturation at specific time points. These events may be altered by air pollutants, which can lead to neuropathological and morphological changes in brain and cause adverse effects on behavioral function (Cory-Slechta et al., 2017).

Recently, increasing evidence shows that air pollutants play significant roles in affecting the development and functions of CNS in children. In heavily polluted urban areas, brains of millions of children are affected by pollutants-derived detrimental effects (Calderon-Garciduenas et al., 2014). Environmental air pollution may induce neurotoxic effects on children (Chiu et al., 2013) and traffic-related air pollution can lead to students’ changes in cognitive development, learning, academic performance or behavior (Sunyer et al., 2015). Studies also suggest the high developmental toxicity of PM_2.5_ which exhibits various harmful effects including structural malformation, growth retardation, dysfunction and death on sexually-matured offspring, due to paternal and/or maternal contact with exogenous physicochemical factors. Furthermore, maternal exposure to environmental particulate pollutants can lead to dysplasia in offspring (Allen et al., 2013; Vrijheid et al., 2016; Li et al., 2017; Veras et al., 2017). Importantly, epidemiological studies suggest an association between childhood autism and air pollutants since children from mothers exposed to high concentration of PM_2.5_ during pregnancy are more likely to be diagnosed with autism (Volk et al., 2011). Notably, childhood autism is related to developmental disorder of hippocampus during embryonic period (Cai et al., 2018), and 11 MRI texture features derived from hippocampus demonstrate the potential to be biomarkers for the diagnosis and characterization of autism spectrum disorders (ASD) (Chaddad et al., 2017). Deficits in spatial working memory have been presented in subjects with autism (Luna et al., 2002; Landa and Goldberg, 2005; Steele et al., 2007). Adult hippocampal neurogenesis has been implicated in cognitive processes and the inhibition of neurogenesis may lead to impaired memory function (Anacker and Hen, 2017). Hippocampus has been regarded as the major brain loci impaired in animals (Chen et al., 2014; Zhang et al., 2016) and subjects with autism (Boucher and Warrington, 1976; Delong, 1978; Delong, 1992), with some acquired hippocampus lesions even demonstrating autistic-like behaviors (Chess, 1971; Delong et al., 1981; Gillberg, 1986). Pregnancy may be a much more sensitive period than childhood because PM_2.5_ can penetrate the maternal air-blood and placental barriers after entering the lung, and thus affect fetal development (Henríquezroldán et al., 2008; Teng et al., 2016). Additionally, early exposure to low-concentration ultrafine particles can lead to persistent CNS functional changes, including impaired long-term learning, short-term memory, impulse-related behavior and motor function (Allen et al., 2014a). In many worldwide regions, individuals may be exposed to particulate air pollutants throughout their lives. Life-long exposure to environmental toxicants for human is difficult to study, especially when environmental factors interact with other common and high-prevalent risk factors such as chronic diseases. Therefore, the real health burden derived from exposure to PM_2.5_ may be underestimated to a large extent.

To the best of our knowledge, it is not well-characterized whether gestational exposure to PM_2.5_ can lead to hippocampus dysplasia in mice offspring. In our preliminary study, gestational exposure to high dosage PM_2.5_ reduces the quantity of neonatal mice offspring by about 48% (*P* < 0.05), compared with the mock-treated group, with some newborn offspring exhibiting deformities at birth (Zhao et al., 2016). Our preliminary work also shows that after gestational exposure to PM_2.5_, the levels of apoptotic proteins in hippocampus of mice offspring of 1-, 7-, 14-, 21-, and 30-day old after birth are all increased, with the most distinct changes demonstrated in 14-day-old offspring which are approximately equivalent to human in childhood period which acts as an important stage of brain development. Thus we preformed the morphological and molecular studies using 14-day-old mice in the present work. Thirty-day-old offspring from mice exposed to PM_2.5_ during pregnancy were subject to the probe test of water maze to evaluate their learning and memory abilities. Collectively, these studies will be helpful to addressing our experimental goal focused on the effects of long-term gestational exposure to PM_2.5_ on hippocampus neurodevelopment in mice offspring and the potential mechanisms.

## Materials and Methods

### Animal Treatment With PM_2.5_

All animal experimental protocols used in the present work were approved by the Animal Experimental Ethics Committee of Weifang Medical University (approval code: 2015266; approval date: December 2015) and conducted according to the guidelines for the Care and Use of Laboratory Animals from National Institutes of Health. Special pathogen- free Kunming mice, 8–9 weeks old, were purchased from Qingdao Animal Experimental Center (Shandong, China) and kept in an air-conditioned room at 25°C, with a 12-h light-dark cycle. The standard laboratory food and water were available all the time. After being adaptively fed for 1 week, female and male mice were crossbred in a ratio of 2:1 and the next day when vaginal plug appeared was designated as day zero of embryonic development (E0). Pregnant mice were randomly divided into five groups (*n* = 6 in each group), namely control, mock-treated, low-dosage, medium-dosage and high-dosage groups. After vaginal plug appeared, pregnant mice were housed in conventional cages, with aspen sawdust, plastic tubing and domes enriched (*n* = 5 per cage). After the last gestational exposure to PM_2.5_, each mouse was housed in a single cage until being raised with its postpartum offspring. Fourteen-day-old mice offspring were randomly selected for morphological and molecular biological analyses, and the remaining sibling offspring were fed for up to 30 days for subsequent MWM test.

The animal model of tracheal drip in pregnant mice was founded according to a method previously described (Zhang et al., 2018). Briefly, the atmospheric PM_2.5_ in winter in a city of Northern China was collected using atmospheric particulate samplers, followed by being freeze-dried for 24 h and preserved at −20°C prior to further analyses. According to the Environmental Air Quality Standard issued by the National Environmental Protection Department of China, the low, medium and high dosages of PM_2.5_ used in the present work were corresponding to the daily average dosage limit of 75 μg/m^3^, the haze red early warning reference value of 500 μg/m^3^ and the highest value of 1000 μg/m^3^ of PM_2.5_ in a certain area in the winter of 2016, respectively.

According to the pulmonary ventilation volume between human and mice, mice from low-, medium- and high-dosage groups were subject to 30 μL PM_2.5_ suspension (PBS as solvent, mixed by ultrasonic oscillation before use) of 0.2592, 1.728, and 3.456 μg/μL, respectively. The mock-treated group was treated using the same volume of PBS, with control group treated with nothing. From the 1st day of pregnancy to delivery, the tracheal drip was performed once every 3 days for a total of 7 times (i.e., performed on E1, E4, E7, E10, E13, E16, and E19, respectively).

### MWM Test

Eight 30-day-old mice offspring from six different pregnant mice in each group were randomly selected for the experiment. Spatial learning and memory were assessed using MWM with the addition of a probe trial performed 3 h after the last learning trial. The MWM using mice offspring was carried out in a black circular tank (diameter as 150 cm and height as 60 cm) (Zheng Hua, Anhui, China) which was divided into four quadrants (east, south, west, and north), with different shape marks as entry points on the pool wall. A 27 cm-high circular platform with a diameter of 8 cm was placed in the target quadrant and the relative locations remained unchanged throughout the experiment, with water level in the pool 0.5 cm higher than the platform. The experiment lasted 7 days and the animals were free to swim 2 min prior to the experiment. The animals were trained twice (60 s once) a day. Animals were put into the water randomly from different entry points and the time needed for animals to find and climb up the platform was recorded. Animals which could not find the platform within 60 s were guided to the platform by the experimenter and the time was recorded as 60 s. On the 7th day, the hidden platform was removed, and the probe test of water maze was performed to record the number of times each mouse passed the hidden platform previously located.

### Ultrastructural Characterization

Six 14-day-old mice offspring from six different pregnant mice in each group were randomly selected for the experiment. The offspring were anesthetized with 5% chloral hydrate. The hippocampus was collected and cut into cubes of about 1 mm^3^ and fixed in 2.5% glutaraldehyde at 4 °C overnight, followed by being fixed in 1% osmium acid for 2 h after being rinsed with PBS. The samples were rinsed using 0.1 M phosphoric acid and dehydrated step by step using ethanol before being embedded and sliced. The ultrastructural changes of neurons were observed using a transmission electron microscope (HT 7700, Hitachi, Tokyo, Japan) after the sections were double-stained using 3% uranium acetate-citric acid.

### EdU Treatment and Immunofluorescence Analysis in the Subgranular Zone (SGZ)

A stock solution of 0.5 mg/mL EdU (Thermo, Logan, UT, United States) was prepared using sterile normal saline (0.9% sodium chloride) as solvent. Based on their respective weight, each mouse was subject to a dosage of 5 mg/kg EdU at a time through intraperitoneal injection twice a day, with continuous administration for 3 days (postnatal days 11–13) and sampling at postnatal day 14.

Six 14-day-old mice offspring from six different pregnant mice in each group were randomly selected for the experiment. After being anesthetized with 5% chloral hydrate, the mice were perfused with 50 mL 0.9% saline and 50 mL 4% paraformaldehyde (0.1 M PBS as solvent, pH = 7.4) successively. After the brains were embedded with OCT compound (Tissue-Tek, Torrance, CA, United States), serial coronal slices (20 μm) were prepared at equal intervals and collected from each of five parallel sets using systematic-random sampling. To quantify neurogenesis, the EdU^+^ cells were analyzed from the complete rostro-caudal extension of the dentate gyrus. One brain hemisphere was randomly selected per animal. After being washed three times using PBS, the slices were placed in 200 mL antigen repair solution (pH = 6.0) for 5-min antigen retrieval in microwave. The slices were blocked using 10% sheep serum after being cooled and washed three times using PBS. Then the slices were incubated with murine monoclonal primary antibodies against NeuN (1:500, Abcam, Cambridge, MA, United States) and GFAP (1:100, CST, Danvers, MA, United States) for immunofluorescence analyses, respectively. The primary antibodies were diluted with 0.1 M PBS, with negative control treated with PBS as well. After being washed using PBS, the slices were incubated with alexa594 anti-mouse IgG (1:200, Invitrogen, Grand Island, NY, United States) at 37°C for 1 h. After being washed using PBS, the slices were then incubated with EdU reaction mixture (Thermo, Logan, UT, United States) at 37 °C for 25 min. Then the slices were incubated with Hoechst mixture (1:1000, Thermo, Logan, UT, United States) at 37°C for 25 min after being washed using PBS, followed by laser confocal microscopic observation (SP8, Leica, Mannheim, Germany) and the quantification was performed by Image-Pro Plus software (version 6.0, Media Cybernetics Inc., Rockville, MD, United States). Five images with high magnification for each sample were selected from each of the five parallel sets of serial slices, respectively. The experimenters were blinded to conditions throughout.

### Terminal Deoxynucleotidyl Transferase dUTP Nick End Labeling (TUNEL) and Double-Immunofluorescence Labeling

Six 14-day-old mice offspring from six different pregnant mice in each group were randomly selected for the experiment. After being anesthetized with 5% chloral hydrate, the offspring were perfused with 50 mL 0.9% saline and 50 mL 4% paraformaldehyde (0.1 M PBS as solvent, pH = 7.4) successively. After perfusion, the brains were collected and immersed in sucrose solution of 20, 25, and 30%, respectively and preserved at 4°C. After the brain was embedded with OCT compound (Tissue-Tek, Torrance, CA, United States), serial coronal slices (20 μm thick) were prepared for immunofluorescence and TUNEL analyses. Slices were collected using systematic-random sampling from each of the five parallel sets. One brain hemisphere was randomly selected per animal. Then the slices were placed in 200 mL antigen repair solution (pH = 6.0) for antigen retrieval by microwave for 5 min after being washed three times using PBS. After being cooled and washed three times using PBS, the slices were blocked using 10% sheep serum. Then the slices were incubated with primary antibodies of murine monoclonal anti-NeuN (1:500, Abcam, Cambridge, MA, United States), murine monoclonal anti-GFAP (1:100, CST, Danvers, MA, United States) and rabbit polyclonal anti-Iba1(1:50, Abcam, Cambridge, MA, United States) respectively to perform the immunofluorescence analyses. The primary antibodies were diluted with 0.1 M PBS. And the negative control was treated with PBS. After being washed using PBS, the slices were incubated with alexa594 anti-mouse IgG (1:200, Invitrogen, Grand Island, NY, United States) or Cy3 anti-rabbit IgG (1:200, Invitrogen, United States) accordingly at 37°C for 1 h. After being washed using PBS, the slices were incubated with TUNEL reaction mixture (50 μL Enzyme solution plus 450 μL label solution) at 37°C for 1 h, with negative control treated with PBS. Then the slices were incubated with Hoechst mixture (1:1000, Thermo, Logan, UT, United States) at 37°C for 25 min after being washed using PBS, followed by analyses using a laser confocal microscopy (SP8, Leica, Mannheim, Germany) and analyzed by Image-Pro Plus software (version 6.0, Media Cybernetics Inc., Rockville, MD, United States). Five images with high magnification for each sample were selected from each of the five parallel sets of serial slices, respectively. The experimenters were blinded to conditions throughout.

### Real-Time Quantitative PCR (RT-qPCR) Analysis

Six 14-day-old mice offspring from six different pregnant mice in each group were randomly selected for the experiment. Total RNA was extracted from the hippocampi of offspring using TRIzol reagent (Invitrogen, Carlsbad, CA, United States) and the relevant cDNA was synthesized using a first-strand cDNA synthesis kit (Roche, Basel, Swiss) according to the supplier’s instructions. The mRNA relative levels were quantified in triplicate by RT-qPCR with SYBR premix (Takara, Otsu, Japan) on a CFX Manager 3.1 system (Bio-Rad, Hercules, CA, United States). Changes in mRNA relative levels were evaluated by the 2^−ΔΔCt^ method. The primers used for RT-qPCR in the present study were presented in Table 1.

**Table 1:** Sequences of primers used for RT-qPCR.

Target	Forward primer (5′–3′)	Reverse primer (5′–3′)
caspase-3	CTGGACTGCGGTATTGAGAC	CCGGGTGCGGTAGAGTAAGC
caspase-8	TGCTTGGACTACATCCCACAC	TGCAGTCTAGGAAGTTGACCA
caspase-9	TCCTGGTACATCGAGACCTTG	AAGTCCCTTTCGCAGAAACAG
Bcl-2	GTCGCTACCGTCGTGACTTC	CAGACATGCACCTACCCAGC
Bax	TGAAGACAGGGGCCTTTTTG	AATTCGCCGGAGACACTCG
p53	GCGTAAACGCTTCGAGATGTT	TTTTTATGGCGGGAAGTAGACTG
c-Fos	CGGGTTTCAACGCCGACTA	TTGGCACTAGAGACGGACAGA
NF-κB	AGGCTTCTGGGCCTTATGTG	TGCTTCTCTCGCCAGGAATAC
TNF-α	ATTCTCTACCCAGCCCCCACTCT	TCCAGGTCACTGTCCCAGCATC
IL-1β	ACCTCACAAGCAGAGCACAAGCC	AAGTCCCTTTCGCAGAAACAG
β-actin	GGCTGTATTCCCCTCCATCG	CCAGTTGGTAACAATGCCATGT

### Western Blotting Analysis

Six 14-day-old mice offspring from six different pregnant mice in each group were randomly selected for the experiment. Total proteins were isolated from the hippocampi of offspring using RIPA lysate and 120 μg proteins for each sample were loaded and separated by SDS-PAGE. Then the proteins were transferred to PVDF membrane (Millipore, Billerica, MA, United States) under 300 mA constant current for 2 h. The membrane was blocked for 2 h using 5% skim milk dissolved in TBST (20 mM Tris-HCl of pH 7.5, 0.5 M NaCl, 0.1% Tween 20, 0.5% Triton X-100) and incubated with the following primary antibodies at 4°C overnight: rabbit anti-caspase-3 polyclonal antibody (1:100, Abcam, Cambridge, MA, United States), rabbit anti-cleaved caspase-3 polyclonal antibody (1:500, CST, Danvers, MA, United States), rabbit anti-cleaved caspase-8 monoclonal antibody (1:300, CST), rabbit anti-cleaved caspase-9 polyclonal antibody (1:500, CST), rabbit anti-Bcl-2 monoclonal antibody (1:500, CST), rabbit anti-Bax polyclonal antibody (1:300, CST), mouse anti-p53 monoclonal antibody (1:500, CST), rabbit anti-c-Fos monoclonal antibody (1:500, CST), mouse anti-GAPDH monoclonal antibody (1:4000, Proteintech, Rosemont, IL, United States) and mouse anti-β-actin monoclonal antibody (1: 3000, MULTI Sciences, Shanghai, China). Then the membrane was washed three times using TBST and incubated with goat anti-IgG (1:2000, Millipore) at room temperature for 2 h. The analyses were conducted with an enhanced chemiluminescence detection kit (Pierce, Rockford, IL, United States) and the band densities of proteins were quantified with ImageJ Gel Analysis tool (National Institutes of Health, Bethesda, MD, United States).

### Enzyme-Linked Immunosorbent Assay (ELISA)

Six 14-day-old mice offspring from six different pregnant mice in each group were randomly selected for the experiment. Proteins from the hippocampi of offspring were extracted using 0.1 M PBS and quantified by an ELISA kit (R&D Systems, Minneapolis, MN, United States) according to the producer’s instructions. Then OD values at 450 nm were detected and concentrations of NF-κB, TNF-α, and IL-1β were calculated based on their standard curves, respectively.

### Statistical Analysis

Experimental data were expressed as mean ± SEM and analyzed using the SPSS19.0 software (SPSS Inc., Chicago, IL, United States). The samples independent *t*-test was used to analyze the ultrastructural and EdU data. The repeated measures two-way ANOVAs combined with a *post hoc* Tukey test were used for the repeated measures of 7 days’ average escape latency analyses in the MWM. All the other data were analyzed by one-way ANOVA followed by a Tukey test. *P*-values less than 0.05 (^∗^*P* < 0.05, ^∗∗^*P* < 0.01) were considered statistically significant.

## Results

### Gestational Exposure to PM_2.5_ Induces Spatial Memory Impairment in Mice Offspring

**FIGURE 1:**
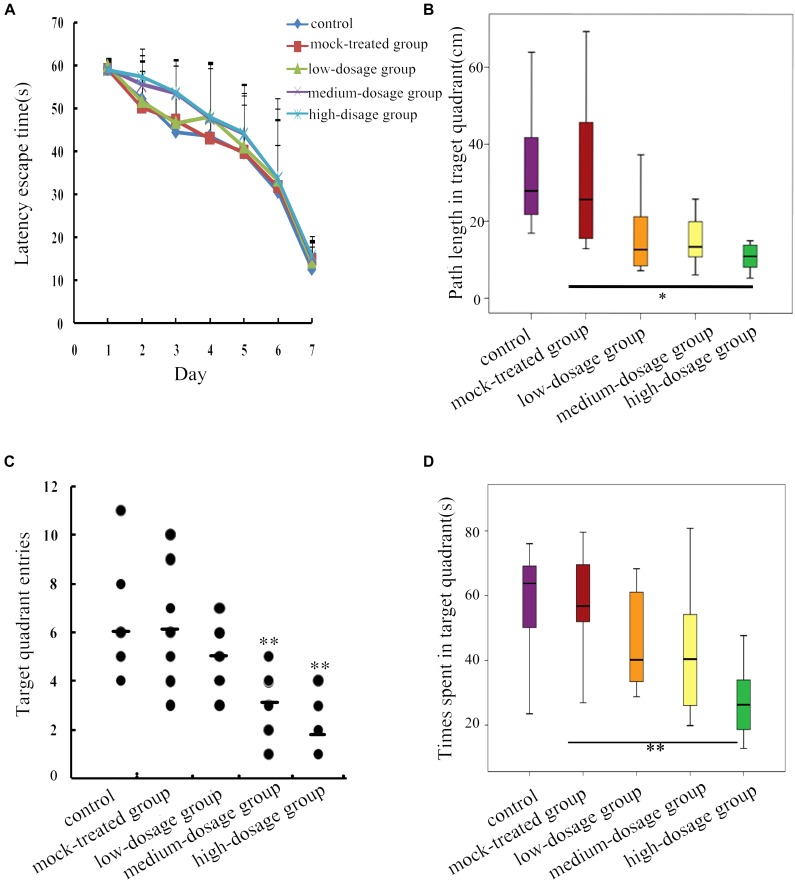
Behavioral changes in 30-day-old mice offspring after gestational exposure to PM_2.5_. **(A)** Changes of escape latency time in directional navigation experiment. **(B)** Swimming distances of mice in target quadrant in the probe test of the water maze (*F* = 4.595, *P* = 0.004). **(C)** Times mice entered the quadrant in the probe test of the water maze (*F* = 8.469, *P* = 0.000). **(D)** Swimming times of mice in target quadrant in the probe test of the water maze (*F* = 4.867, *P* = 0.003) (*n* = 8).

Thirty-day-old mice offspring were subject to MWM to evaluate the effects of PM_2.5_ exposure on their spatial learning and memory abilities. Changes of escape latency time in 7-day directional navigation experiment were shown (Figure 1A). We found that the escape latency time was gradually shortened after learning and training despite of no significant difference between the PM_2.5_- and mock-treated groups (*P* > 0.05). Furthermore, compared with the mock-treated group, high-dosage group exhibited significant decrease in the distance mice traveled within the target quadrant in the probe test of water maze (*P* < 0.05) (Figure 1B). And mice offspring from both medium- and high-dosage groups presented significantly decreased numbers of crossing the target quadrant, in comparison with the mock-treated group (*P* < 0.01) (Figure 1C). The movement time within the target quadrant for offspring from high-dosage group was significantly shorter than that from the mock-treated group (*P* < 0.01) (Figure 1D). Collectively, our results show that gestational exposure to PM_2.5_, particularly at high dosage, affects neurobehavioral functions and induces spatial memory impairment in mice offspring.

### Gestational Exposure to PM_2.5_ Impairs Ultrastructure of Hippocampal Neurons in Mice Offspring

**FIGURE 2:**
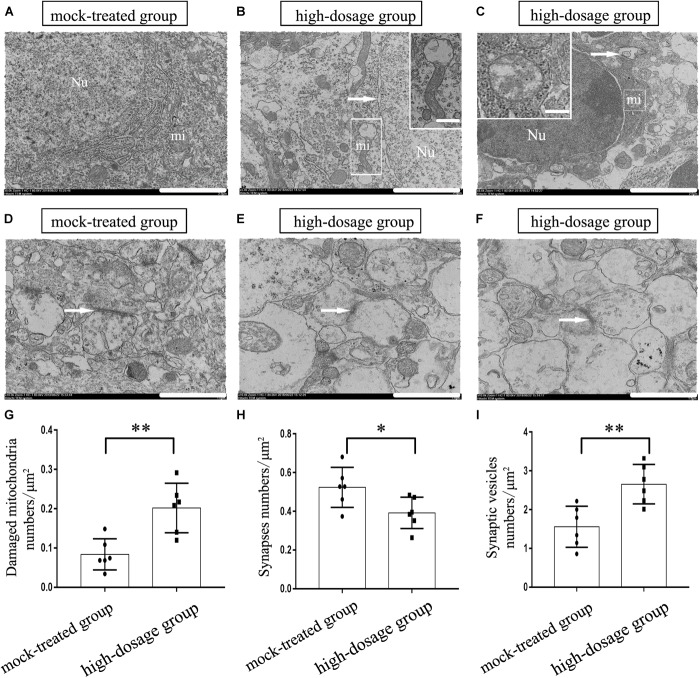
Effects of gestational exposure to PM_2.5_ on the ultrastructure of neurons in 14-day-old mice offspring. **(A–C)** Neurons in mock-treated and high-dosage groups (Magnification is 5000×, bar = 2 μm, inset bar = 0.2 μm), the arrow shows the nucleus gap widening in **(B)** and autophagosome in **(C)**, Neurons in high-dosage group displayed partial vagueness in mitochondrial cristae, vacuolar degeneration in mitochondrion. **(D–F)** Synapses in mock-treated and high-dosage groups (Magnification is 10^4^×, bar = 1 μm). Nu, nucleus; mi, mitochondrion. **(G)** Numbers of damaged mitochondria statistical analyses per μm^2^ (*t* = 3.887, *P* = 0.003). **(H)** Numbers of synapses statistical analyses per μm^2^ (*t* = 2.455, *P* = 0.034). **(I)** Numbers of synapses vesicles statistical analyses per μm^2^ (*t* = 3.663, *P* = 0.003). ^∗^*P* < 0.05, ^∗∗^*P* < 0.01, compared with mock-treated group (*n* = 6).

The modulation of spatial memory can be mediated by hippocampal newborn neurons (Zhou et al., 2017). To explore the structural basis of spatial memory impairment in mice offspring, we further evaluated the ultrastructural changes of hippocampal neurons. We characterized ultrastructural properties in hippocampal CA1, CA3, and DG regions of 14-day-old mice offspring and found that hippocampal neurons in the mock-treated group exhibited normal morphology, abundant mitochondria and complete nuclear membrane (Figure 2A). In contrast, those in the high-dosage group demonstrated the most significant changes in the CA3 region. The mitochondrial swelling was mainly matrix-type. Partial vagueness in mitochondrial cristae, vacuolar degeneration in mitochondrion and widened nuclear perinuclear gap in hippocampal neurons of mice offspring from high-dosage group were observed (Figures 2B,C). Moreover, gestational exposure to PM_2.5_ also led to similar cellular defects in ultrastructure of cortical neurons (Supplementary Figure S1). Severe structural and functional alterations in hippocampal cells, specifically in the mitochondria, were demonstrated after adolescent binge-like alcohol exposure, affecting adult brain memory function (Tapia-Rojas et al., 2017). Therefore, the impaired mitochondria in hippocampus may be one of the reasons for the decline of spatial memory in mice offspring.

The molecular mechanisms of memory consolidation and long-term storage are reported dating from synaptic level, and both preexisting synapses remodeling and changes of synapses number may play a vital role in information expression and storage (Bailey et al., 2015). Then, we investigated the ultrastructure of neuronal synapses. As is shown in Figure 2D, the synapses are asymmetrical ones (excitatory ones) based on the ultrastructure and the mock-treated group displayed synapses with distinct structures, including evidently-shown synaptic vesicles and synaptic gap. Differently, the synaptic structures of hippocampi from PM_2.5_-treated group, especially from the high-dosage group, were significantly changed. Notably, we observed fuzzy synaptic structure, increased presynaptic vesicles, thickened postsynaptic compacts and decreased synaptic space in high-dosage group (Figures 2E,F). Lastly, the numbers of mitochondria, synapses and synaptic vesicles per unit area were analyzed and the high-dosage group showed significantly increased damaged mitochondria (*P* < 0.01) and synaptic vesicles (*P* < 0.01) compared with the mock-treated group, while the amount of synapses decreased (*P* < 0.05) (Figures 2G–I). So we speculate the decrease of excitatory synapses and widening of synaptic gap in hippocampal neurons may affect the transmission of nerve impulses and consequently the storage of memory. In summary, gestational exposure to PM_2.5_ may impair the structure of mitochondria and synapses in hippocampal neurons in mice offspring, which may be the structural basis for the decline in spatial memory. Then, the potential mechanism was investigated.

### Gestational Exposure to PM_2.5_ Affects the Neurogenesis of SGZ in Mice Offspring

**FIGURE 3:**
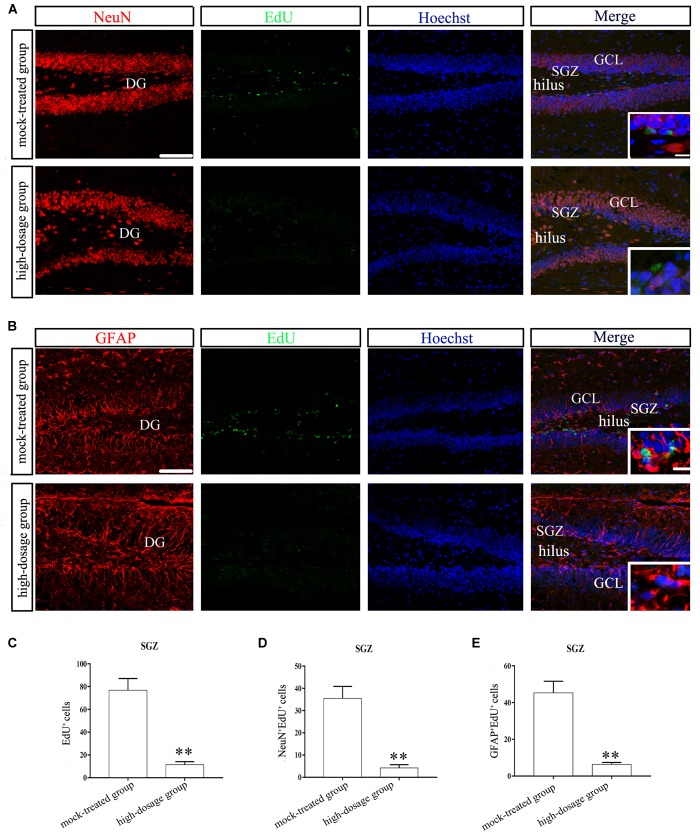
Neurogenesis in hippocampal SGZ region of 14-day-old mice offspring after exposure to PM_2.5_. **(A,B)** Laser confocal photographs of NeuN/EdU and GFAP/EdU labeling in hippocampal DG regions, respectively. NeuN staining (red), GFAP staining (red), EdU staining (green) and nuclear staining (Hoechst, blue). Magnification is 400×, bar = 75 μm, inset bar = 7.5 μm. **(C)** EdU^+^ cells (*t* = 15.025, *P* = 0.000). **(D)** NeuN^+^/EdU^+^cells (*t* = 13.726, *P* = 0.000). **(E)** GFAP^+^/EdU^+^cells (*t* = 13.759, *P* = 0.000). ^∗∗^*P* < 0.01, compared with mock-treated group (*n* = 6).

To quantify neurogenesis in DG, we analyzed the number of EdU-positive cells using confocal microscopy. NeuN/EdU and GFAP/EdU double-labeling were performed to explore the effects of gestational exposure to PM_2.5_ on neuronal progenitors in the SGZ. First, we found that the total number of EdU-positive cells in the SGZ was significantly reduced due to high-dosage PM_2.5_ exposure (*P* < 0.01) (Figures 3A–C). NeuN^+^/EdU^+^ and GFAP^+^/EdU^+^ double-labeled cells were significantly decreased in high-dosage group compared to those in the mock-treated group (*P* < 0.01) (Figures 3D,E). Thus, gestational exposure to PM_2.5_ affects the neurogenesis in the SGZ of mice offspring.

### Gestational Exposure to PM_2.5_ Increases Apoptosis of Hippocampal Cells in Mice Offspring

**FIGURE 4:**
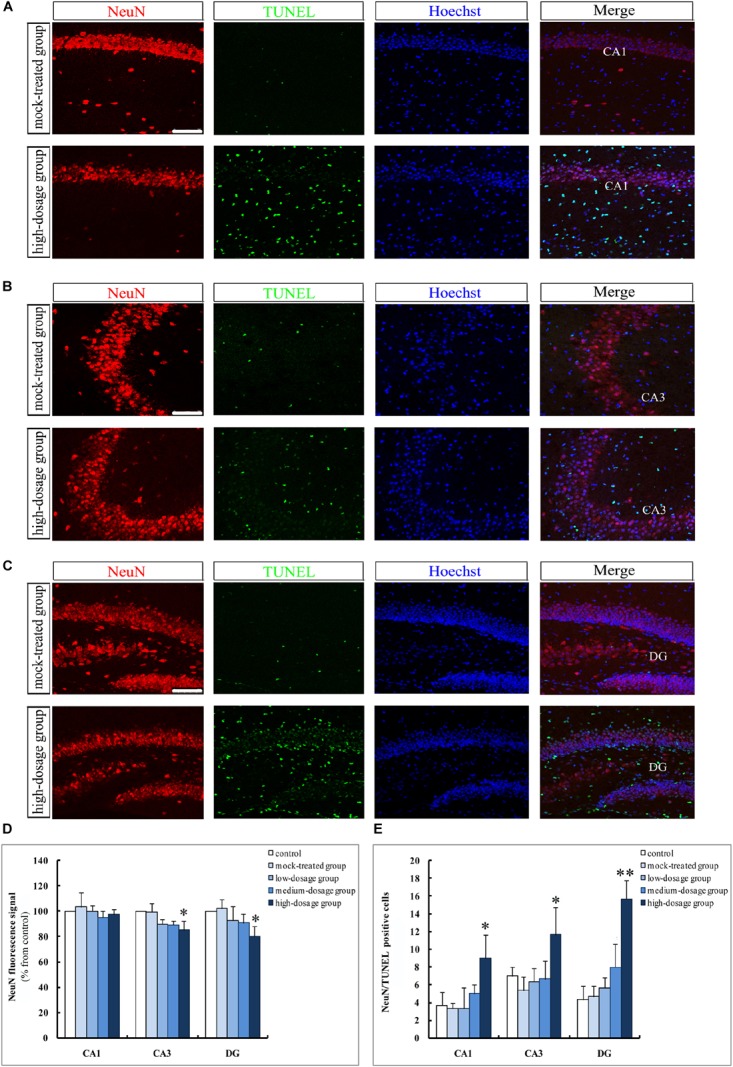
Apoptosis of hippocampal neurons in 14-day-old mice offspring after exposure to PM_2.5_. **(A–C)** Laser confocal photographs of NeuN^+^/TUNEL^+^ in hippocampal CA1, CA3, and DG regions, respectively. NeuN staining (red), TUNEL staining (green) and nuclear staining (Hoechst, blue). Magnification is 400×, bar = 75 μm. **(D)** NeuN fluorescence intensity as marker of neurons (*F* = 0.357, *P* = 0.833, see CA1; *F* = 5.368, *P* = 0.014, see CA3; *F* = 4.037, *P* = 0.033, see DG). **(E)** NeuN^+^/TUNEL^+^ cells (*F* = 5.448, *P* = 0.014, see CA1; *F* = 4.716, *P* = 0.021, see CA3; *F* = 20.255, *P* = 0.000, see DG). ^∗^*P* < 0.05, ^∗∗^*P* < 0.01, compared with mock-treated group (*n* = 6).

**FIGURE 5:**
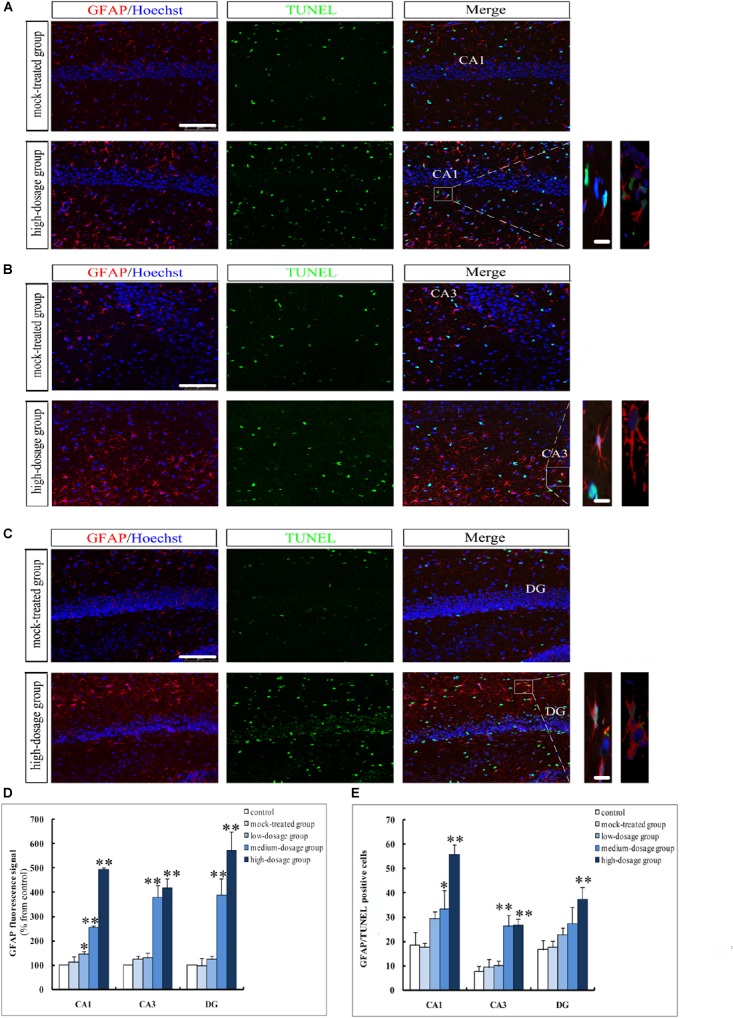
Apoptosis of hippocampal astrocytes in 14-day-old mice offspring after exposure to PM_2.5_. **(A–C)** Laser confocal photographs of GFAP^+^/TUNEL^+^ in hippocampal CA1, CA3, and DG regions, respectively. GFAP staining (red), TUNEL staining (green) and nuclear staining (Hoechst, blue). Magnification is 400×, bar = 75 μm, inset bar = 10 μm. **(D)** GFAP fluorescence intensity as marker of reactive astrocytes (*F* = 511.345, *P* = 0.000, see CA1; *F* = 53.672, *P* = 0.000, see CA3; *F* = 58.273, *P* = 0.000, see DG). **(E)** GFAP^+^/TUNEL^+^cells (*F* = 30.690, *P* = 0.000, see CA1; *F* = 30.643, *P* = 0.000, see CA3; *F* = 10.686, *P* = 0.001, see DG). ^∗^*P* < 0.05, ^∗∗^*P* < 0.01, compared with mock-treated group (*n* = 6).

**FIGURE 6:**
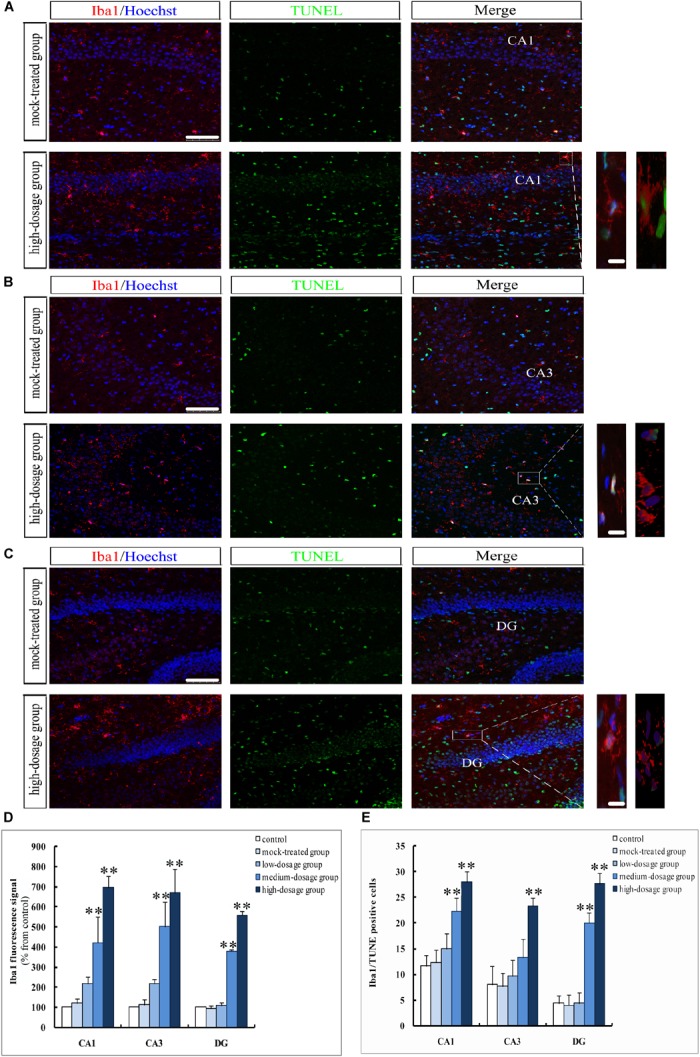
Apoptosis of hippocampal microglia in 14-day-old mice offspring after exposure to PM_2.5_. **(A–C)** Laser confocal photographs of Iba1^+^/TUNEL^+^ in hippocampal CA1, CA3, and DG regions, respectively. Iba1 staining (red), TUNEL staining (green) and nuclear staining (Hoechst, blue). Magnification is 400×, bar = 75 μm, inset bar = 10 μm. **(D)** Iba1 fluorescence intensity as marker of reactive microglia (*F* = 42.666, *P* = 0.000, see CA1; *F* = 30.772, *P* = 0.000, see CA3; *F* = 738.626, *P* = 0.000, see DG). **(E)** Iba1^+^/TUNEL^+^cells (*F* = 24.989, *P* = 0.000, see CA1; *F* = 14.350, *P* = 0.000, see CA3; *F* = 96.904, *P* = 0.000, see DG). ^∗^*P* < 0.05, ^∗∗^*P* < 0.01, compared with mock-treated group (*n* = 6).

In another set of experiments, we addressed the question that whether gestational exposure to PM_2.5_ affects apoptosis of hippocampal neurons, astrocytes and microglia in mice offspring. First, NeuN fluorescence intensities of neurons showed no significant difference within the CA1 region among different groups (*P* > 0.05). However, CA3 and DG regions from the high-dosage group demonstrated significantly lower NeuN fluorescence intensities compared to the mock-treated group (*P* < 0.05) (Figures 4A–D). Furthermore, the number of NeuN^+^/TUNEL^+^ double-labeled cells was significantly increased in high-dosage group compared to that in the mock-treated group (0.01 < *P* < 0.05 for CA1 and CA3 regions, *P* < 0.01 for DG region) (Figure 4E). Immunofluorescence intensities of astrocytes and microglia in pyramidal layers and stratum radiatum of CA1 and CA3 regions and the granule cell layer of the DG were increased with PM_2.5_ exposure dosage (Figures 5, 6).

The GFAP- and Iba1-positive cells in the hippocampus of mice offspring from PM_2.5_-treated groups increased gradually with exposure dosage, with cell body hypertrophic and protuberance increased and prolonged. The immunofluorescence intensities of GFAP and Iba1 in CA1, CA3, and DG regions from medium- and high-dosage groups (*P* < 0.01), along with that of GFAP in CA1 region from low-dosage group (0.01 < *P* < 0.05), significantly increased, suggesting an activation of hippocampal neuroglial cells in mice offspring after gestational exposure to PM_2.5_ (Figures 5, 6A–D). Meanwhile, the numbers of GFAP^+^/TUNEL^+^ and Iba1^+^/TUNEL^+^ double-labeled cells also increased with the PM_2.5_ exposure. The numbers of GFAP^+^/TUNEL^+^ and Iba1^+^/TUNEL^+^ double-labeled cells in CA1, CA3 and DG regions from high-dosage group, GFAP^+^/TUNEL^+^ double-labeled cells in CA1 and CA3 regions and Iba1^+^/TUNEL^+^ double-labeled cells in CA1 and DG regions from medium-dosage group were significantly higher than those of the mock-treated group (*P* < 0.01 for high-dosage group, *P* < 0.01 for medium-dosage group while the GFAP^+^/TUNEL^+^ double-labeled cells in CA1 region as 0.01 < *P* < 0.05), suggesting increased apoptosis of astrocytes and microglia (Figures 5, 6A–C,E).

### Gestational Exposure to PM_2.5_ Alters Expressions of Apoptosis-Related Genes in Hippocampus of Mice Offspring

**FIGURE 7:**
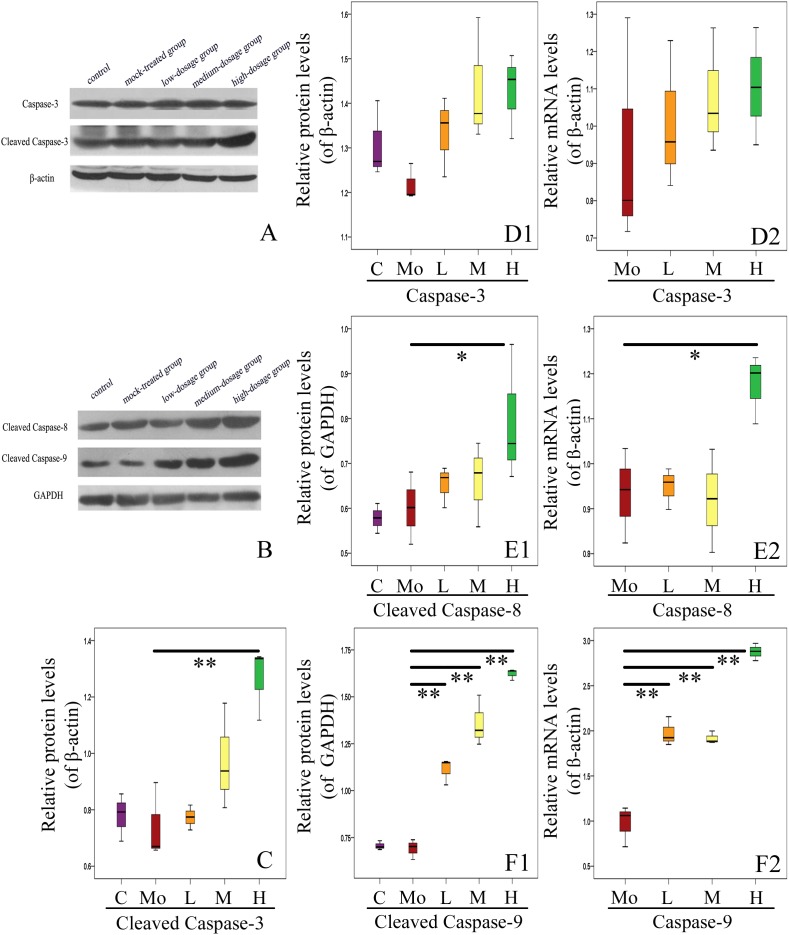
Effects of gestational exposure to PM_2.5_ on hippocampal apoptosis pathways in 14-day-old mice offspring. **(A,C,D1)** Protein relative level changes of caspase-3 and cleaved caspase-3 (*F* = 9.248, *P* = 0.002, see C; *F* = 2.642, *P* = 0.097, see **D1**). **(D2)** mRNA relative level changes of caspase-3 (*F* = 0.359, *P* = 0.832). **(B,E1,F1)** Protein relative levels of cleaved caspase-8 and cleaved caspase-9, respectively (*F* = 4.308, *P* = 0.028, see **E1**; *F* = 90.682, *P* = 0.000, see **F1**). **(E2,F2)** mRNA relative level changes of caspase-8 and caspase-9, respectively (*F* = 5.144, *P* = 0.016, see **E2**; *F* = 103.186, *P* = 0.000, see **F2**). All protein and mRNA bands were expressed as ratio of β-actin or GAPDH, respectively. Values were presented as mean ± SEM. ^∗^*P* < 0.05, ^∗∗^*P* < 0.01 (*n* = 6).

Next we analyzed the mechanisms of apoptosis in hippocampus of mice offspring. Western blotting analysis and RT-qPCR of caspase-3 and cleaved caspase-3 were performed (Figure 7). We found no significant difference between the PM_2.5_- and mock-treated groups in the levels of mRNA and protein of caspase-3 (*P* > 0.05) (Figures 7A,D1,D2). Specifically, Western blotting analysis was carried out using an antibody capable to detect endogenous levels of the large fragments of activated caspase-3 without recognizing the full length caspase-3 or other cleaved caspases. Highly expressed cleaved caspase-3 in the hippocampus of mice can be learned (Figure 7A). High-dosage group exhibited significantly higher level of cleaved caspase-3 than the mock-treated group (*P* < 0.01) (Figure 7C).

We then checked the activation of apoptosis-related caspases by analyzing the expressions of caspase-8 and caspase-9. The relative levels of caspase-8 mRNA and cleaved caspase-8 protein in hippocampus of mice offspring from high-dosage group increased significantly, compared with the mock-treated group (0.01 < *P* < 0.05) (Figures 7B,E1,E2). Caspase-9 is an important participator in mitochondrial apoptosis pathway and can play a role in apoptosis by activating caspase-3. The relative levels of caspase-9 mRNA and cleaved caspase-9 protein in hippocampus of mice offspring from all experimental groups were significantly up-regulated with increased PM_2.5_ exposure dosage (*P* < 0.01) (Figures 7B,F1,F2). Our results strongly support the important roles of caspase family proteins in mediating the apoptosis in hippocampus of mice offspring after gestational exposure to PM_2.5_.

**FIGURE 8:**
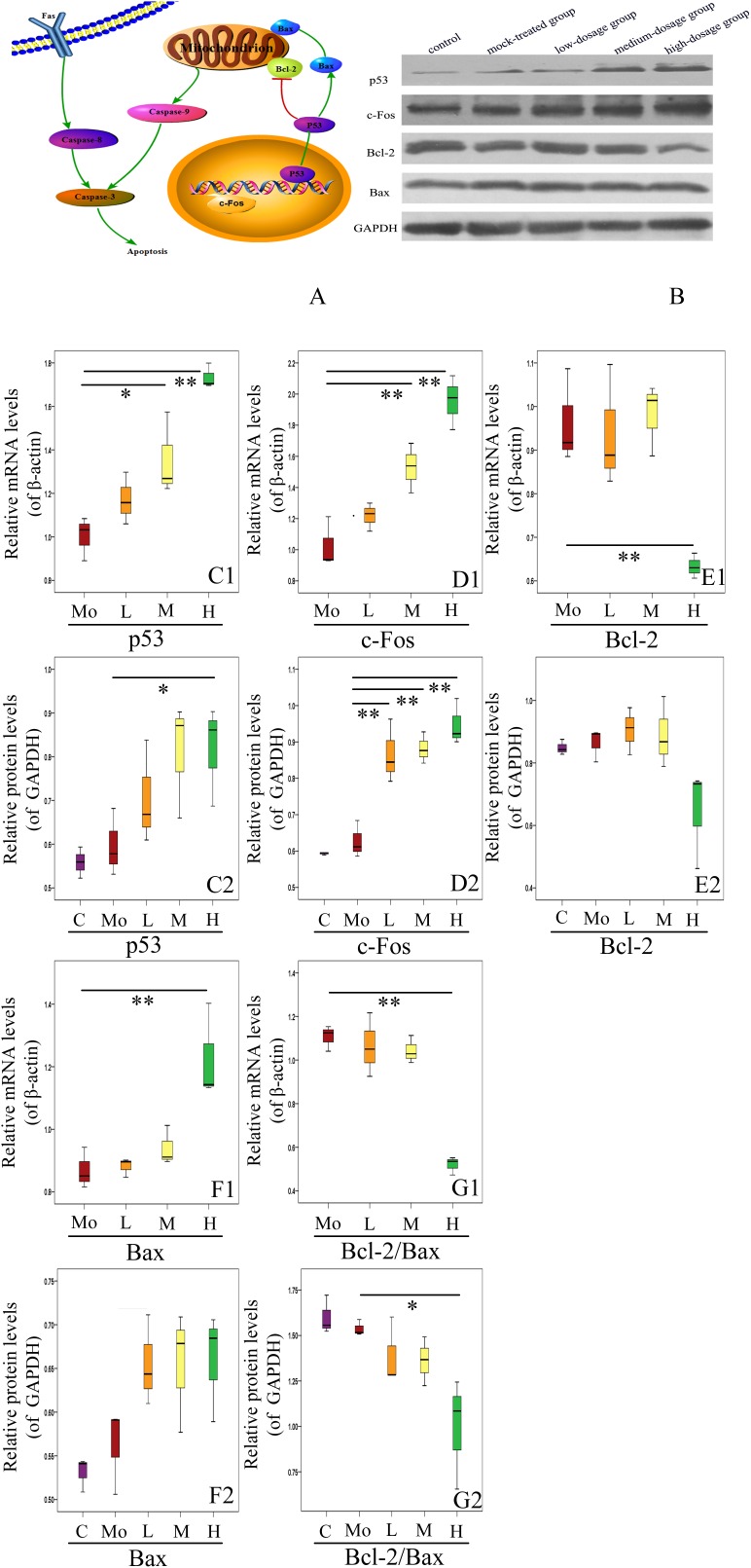
Effects of gestational exposure to PM_2.5_ on apoptotic pathways in hippocampus of 14-day-old mice offspring. **(A)** Schematic of the mechanism of hippocampal apoptosis induced by gestational exposure to PM_2.5_. **(B)** Representative western blotting. **(C1–G2)** Protein and mRNA relative level changes of p53, c-Fos, Bcl-2, Bax, and Bcl-2/Bax ratio, respectively (*F* = 21.924, *P* = 0.000, see **C1**; *F* = 4.110, *P* = 0.032, see **C2**; *F* = 26.763, *P* = 0.000, see **D1**; *F* = 24.227, *P* = 0.000, see **D2**; *F* = 8.924, *P* = 0.002, see **E1**; *F* = 3.538, *P* = 0.048, see **E2**; *F* = 9.725, *P* = 0.002, see **F1**; *F* = 3.963, *P* = 0.035, see **F2**; *F* = 28.754, *P* = 0.000, see **G1**; *F* = 5.285, *P* = 0.015, see **G2**). All protein and mRNA bands were expressed as ratio of β-actin or GAPDH, respectively. Values were presented as mean ± SEM. ^∗^*P* < 0.05, ^∗∗^*P* < 0.01 (*n* = 6).

Furthermore, we investigated the expressions of other apoptosis-related genes (Figure 8A). We found that the mRNA level of Bax in hippocampus of mice offspring was enhanced with PM_2.5_ exposure dosage, with that in high-dosage group significantly higher than the mock-treated group (*P* < 0.01). There was no significant difference in the protein level of Bax between the PM_2.5_- and mock-treated groups (*P* > 0.05) (Figures 8B,F1,F2). Additionally, the mRNA level of Bcl-2 in high-dosage group was significantly lower than that in the mock-treated group (*P* < 0.01), and the protein level of Bcl-2 did not change between the PM_2.5_- and mock-treated groups (Figures 8B,E1,E2). The balance between Bcl-2 and Bax is closely related to cell apoptosis (Wang et al., 2015). The Bcl-2/Bax ratios of both mRNA and protein relative levels in hippocampus of mice offspring from high-dosage group were significantly decreased, resulting in cell apoptosis (*P* < 0.01 for mRNA relative levels, 0.01 < *P* < 0.05 for protein relative levels) (Figures 8G1,G2). Furthermore, c-Fos mRNA levels from medium- and high-dosage groups exhibited significant increases (*P* < 0.01), and c-Fos protein levels from all experimental groups were significantly enhanced (*P* < 0.01), compared to the mock-treated group. Lastly, p53 presented significantly higher mRNA levels in medium- (0.01 < *P* < 0.05) and high-dosage groups (*P* < 0.01) and protein relative level in high-dosage group (0.01 < *P* < 0.05), when compared to the mock-treated group, respectively (Figures 8B,C1,C2,D1,D2). Therefore, gestational exposure to PM_2.5_ significantly affects the expressions of apoptosis-related genes in hippocampus of mice offspring, in line with their neurobehavioral changes.

### Gestational Exposure to PM_2.5_ Induces Inflammation in Hippocampus of Mice Offspring

**FIGURE 9:**
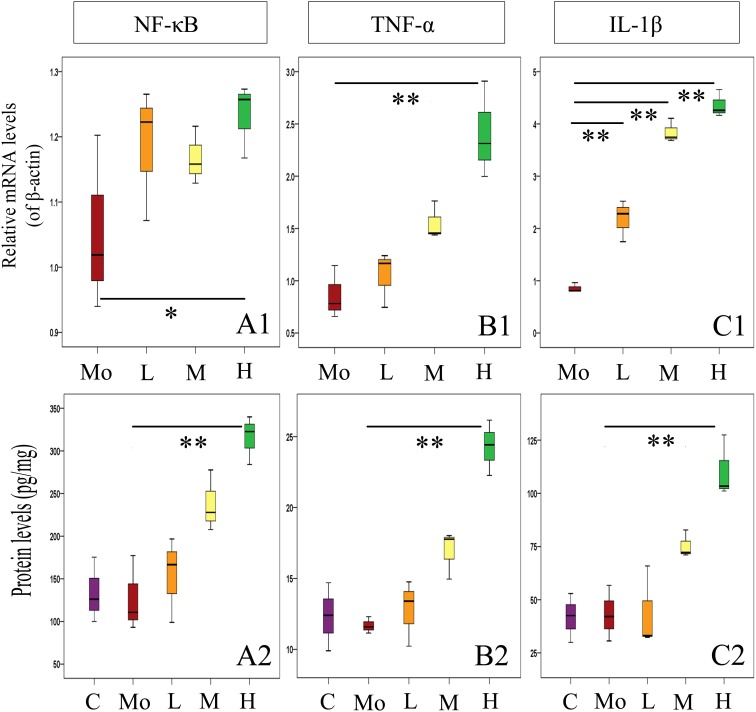
Effects of gestational exposure to PM_2.5_ on the expressions of inflammatory factors in hippocampus of mice offspring. **(A1–C2)** Protein and relative mRNA level changes of NF-κB, TNF-α, and IL-1β, respectively (*F* = 4.214, *P* = 0.030, see **A1**; *F* = 12.257, *P* = 0.001, see **A2**; *F* = 15.704, *P* = 0.000, see **B1**; *F* = 22.841, *P* = 0.000, see **B2**; *F* = 134.631, *P* = 0.000, see **C1**; *F* = 14.738, *P* = 0.000, see **C2**). All mRNA bands were expressed as ratio of β-actin, respectively. Values were presented as mean ± SEM. ^∗^*P* < 0.05, ^∗∗^*P* < 0.01 (*n* = 6).

Subsequently, we investigated the effects of gestational exposure to PM_2.5_ on the levels of inflammatory molecules in hippocampus of mice offspring. Specifically, NF-κB, TNF-α, and IL-1β were evaluated by RT-qPCR and ELISA (Figure 9). We found that the mRNA levels of NF-κB, TNF-α, and IL-1β were increased with PM_2.5_ exposure dosage. When compared to the mock-treated group, mRNA levels of IL-1β from the experimental groups increased. Additionally, the mRNA levels of TNF-α (*P* < 0.01) and NF-κB (0.01 < *P* < 0.05) from high-dosage group were significantly up-regulated compared to the mock-treated group, respectively. Meanwhile, the protein levels of NF-κB, TNF-α, and IL-1β increased positively with the PM_2.5_ exposure dosage and were significantly enhanced in high-dosage group in comparison with the mock-treated group (*P* < 0.01) (Figures 9A1–C2). Collectively, our results strongly suggest that gestational exposure to PM_2.5_ can induce hippocampus inflammation in mice offspring.

## Discussion

In this study, we investigated the potential effects of gestational exposure to PM_2.5_, a representative form of air pollutants, on hippocampus development in mice offspring via neurobehavioral, ultrastructural, biochemical and molecular studies. Air pollution is a worldwide environmental health problem and pollutants exposed during pregnancy might accumulate in offspring (Satoshi et al., 2016). Epidemiological evidences show that exposure to particulate air pollutants during development period affects brain development in offspring (Chiu et al., 2016). Although the air quality in many countries has been improved significantly, PM_2.5_ still acts as one of the most important air pollutants to which pregnant women are more likely to be exposed. In view of the unknown effects of gestational exposure to PM_2.5_ on the neurodevelopment in mice offspring and the potential mechanisms, *in vivo* systematic researches are urgently needed to understand its effects on hippocampus development in mice offspring.

The present work clearly demonstrated the detrimental effects induced by gestational exposure to PM_2.5_ on mice offspring, including impaired spatial memory ability. Fetal brain development, extremely susceptible to toxic substances, may be readily hindered by intrauterine exposure to air pollutants which may exert long-term and profound effects on neurodevelopment and neurofunctions in offspring (Kelly and Fussell, 2015). Some studies have shown that structural changes in hippocampus may result in depression and anxiety (Sapolsky, 2000) and be associated with motor behavior deficit related to autism (Bergeron et al., 2013). The ultrastructure of hippocampal neurons in mice offspring exposed to PM_2.5_ showed obvious mitochondrial damage, fuzzy synaptic structure, increased presynaptic vesicles, thickened postsynaptic compacts, decreased synaptic space, and decreased number of asymmetrical synapses especially. Neuronal and synaptic ultrastructure changes may serve as the morphological basis for spatial memory damage caused by aluminum exposure (Zhang et al., 2013). In the present work, gestational exposure to high concentrations of PM_2.5_ destroys the structure of mitochondria and synapses in hippocampal neurons, affecting the information transmission function of neurons and leading to the decline of spatial memory ability in mice offspring. Further investigations are needed for the definite mechanism by which gestational exposure to PM_2.5_ exerts effects on the neurodevelopment in mice offspring, however.

Inhibition of neurogenesis results in impaired acquisition of spatial relational memory which calls for the encoding and flexible utilization of positional relations between cues (Dupret et al., 2008). The ability to acquire new memories in adulthood may depend on neurogenesis which acts as the basis of brain memory (Deng et al., 2010). Adult neurogenesis, involved in new neurons’ birth, survival and functional integration into existing neural circuitries, is limited to two regions of subgranular zone (SGZ) and subventricular zone (SVZ) in rodents (Alvarez-Buylla et al., 2001). Based on our preliminary findings, we hypothesized that gestational exposure to PM_2.5_ would inhibit the neurogenesis in hippocampus of mice offspring, which was confirmed in the present work. In the SGZ of mice offspring, the total numbers of both EdU^+^ and NeuN^+^/EdU^+^ cells were significantly decreased due to high-dosage gestational exposure to PM_2.5_ which may exert effects on the birth of new neurons in the SGZ of offspring. The decrease in survival, differentiation, and maturation of newborn neurons results in impaired short-term and long-term spatial memory (Bassani et al., 2017). The retrieval of new and remote episodic memory is impaired due to reduced adult neurogenesis in depression (Fang et al., 2018). And the impaired adult hippocampal neurogenesis may underlie the cognitive deficits observed in depression (Sahay and Hen, 2007). Diesel exhaust exposure results in impaired cellular proliferation in the SVZ in males and reduced adult neurogenesis, with male mice exhibiting fewer new neurons in the SVZ (Coburn et al., 2018). To the best of our knowledge, there has been no report regarding the association between exposure to PM_2.5_ and the impaired neurogenesis in the SVZ niche.

Neurogenesis, constantly occurring in adult brain, decreases with age (Ming and Song, 2011), probably due to the increased neuroinflammation and microglial activation (Schuitemaker et al., 2012). The present work also suggests that gestational exposure to PM_2.5_ impairs neurogenesis by a mechanism likely to involve microglia activation and neuroinflammation. Neuroinflammation, oxidative stress or activation of neuroglial cells have been regarded as presumed mechanisms for the possible damages to human CNS induced by PM_2.5_ exposure (Allen et al., 2014b). One of the biological mechanisms by which air pollutants may affect brain is through neuroinflammation (Allen et al., 2013). Exposure to PM_2.5_ causes chronic inflammation in pregnant women, along with retarded fetal development and genetic changes in fetal hippocampus (Chao et al., 2017). Our results showed that the exposure led to activation of microglia and astrocytes, along with enhanced GFAP-labeled astrocytes and Iba1-labeled microglia in CA1, CA3, and DG regions, suggesting the activation of neuroglial cells in these hippocampal regions. Thus, it can be speculated that gestational exposure to PM_2.5_ can activate microglia and astrocytes in the hippocampus of mice offspring, resulting in the release of proinflammatory factors (Chew et al., 2013; Baburamani et al., 2014). This is consistent with the findings that abnormal development or function of astrocytes at different developmental stages (such as fetal and postnatal ones) might lead to different neurodevelopmental diseases (Molofsky et al., 2012; Freeman and Rowitch, 2013). It has been shown that the release of TNF-α and IL-6 is increased after the activation of microglia (Gresa-Arribas et al., 2012). In the present study, the protein and relative mRNA levels of inflammatory factors of NF-κB, TNF-α, and IL-1β were increased with the exposure dosage. Notably, microglia in the hippocampus may be more vulnerable to dysfunction or apoptosis (Shigemoto-Mogami et al., 2014). As reported by Allen et al. (2014b), the variation pattern of glial in female brain is presented as transient astroglia reaction, with that of male brain as dysfunction of microglia and stellate cells till early adulthood. NF-κB is a redox-sensitive transcription factor that can convert oxidative stress signals into expression changes of the genes (such as TNF-α and IL-1β) related to the activities of different cells (Lin et al., 2011; Atala, 2012). Mitochondrial perturbation can induce the apoptosis and release of apoptosis factors. TNF-α and IL-1β, two important proinflammatory cytokines, could regulate synaptic plasticity in hippocampus and activate astrocytes related to synaptic plasticity (Balschun et al., 2004). These inflammatory factors can serve as a bridge between neuroinflammation and excitotoxicity, which exerts critical effects on the hippocampus development in mice offspring.

Neuronal apoptosis induced by over-activation of microglia during neuroinflammation has been reported conducive to the pathology of CNS degenerative diseases (Feng et al., 2016), thus we speculate exposure to PM_2.5_ may also induce apoptosis as well as neuroinflammation and glial cell activation, and the expressions of apoptosis-related genes in hippocampus of mice offspring were investigated subsequently. Prenatal exposure to air pollution has been associated with ASD risk. Apoptosis plays an important role in the pathogenesis of autism (Dong et al., 2018). Co-exposure to PM_2.5_, SO_2_ and NO_2_ impairs spatial learning and memory abilities and causes abnormal expressions of apoptosis-related genes (Ku et al., 2016). In the present work, the GFAP^+^/TUNEL^+^ and Iba1^+^/TUNEL^+^ cells counting suggested the increased apoptosis of neuroglial cells with increased exposure dosage. With the increase of PM_2.5_ exposure dosage, the apoptotic rate of hippocampal cells and the number of TUNEL-positive neurons were up-regulated, suggesting that gestational exposure to PM_2.5_ altered neural development in mice offspring through apoptosis. Therefore, the apoptosis-related proteins, caspase-3, caspase-8, and caspase-9 which can control the endogenous and exogenous pathways of apoptosis, were further analyzed. As a transcription factor, p53 can regulate the expressions of many genes related to cell cycle progression, apoptosis, DNA repair and stress response (Goshen and Yirmiya, 2009). C-Fos is also a transcription factor and can regulate many cellular processes. The balance between Bcl-2 and Bax determines whether apoptosis occurs. In the present study, the protein expressions of p53, c-Fos, and Bax were increased while Bcl-2 was decreased. Collectively, our results suggest that gestational exposure to PM_2.5_ may regulate the apoptotic factors that can induce neuronal apoptosis through activating endogenous and exogenous apoptotic pathways, including the mitochondrial pathway in mice offspring.

Our results demonstrate the importance of hippocampus and how PM_2.5_ may affect fetal brain development. Particularly, gestational exposure to PM_2.5_ induces hippocampal cell apoptosis, neuroinflammation, neuroglial activation, and neurogenesis impairment, associated with altered hippocampus development and impaired neurobehavioral functions in mice offspring. These findings may be helpful to provide insights to identify therapeutic targets to attenuate the disorders in human induced by exposure to PM_2.5_ or other air pollutants.

## Author Contributions

LY and YG designed the experiments, interpreted the results, and critically revised the manuscript. XZ and XW performed the experiments and wrote the manuscript. TW, HZ, and HW analyzed the data and generated figures. CZ performed the interpretation of data for the work and revised the manuscript.

## Conflict of Interest Statement

The authors declare that the research was conducted in the absence of any commercial or financial relationships that could be construed as a potential conflict of interest.

## Abbreviations

CNS: central nervous system
MWM: Morris water maze
PBS: phosphate buffered solution
PM_2.5_: particulate matter 2.5
RT-qPCR: real-time quantitative PCR
TUNEL: terminal deoxynucleotidyl transferase dUTP nick end labeling.
