# Supplementary material for: Gestational Exposure to Particulate Matter 2.5 (PM2.5) Leads to Spatial Memory Dysfunction and Neurodevelopmental Impairment in Hippocampus of Mice Offspring
Source: Front Neurosci. 2019 Jan 7;12:1000. doi: 10.3389/fnins.2018.01000 (PMC6330280; doi:10.3389/fnins.2018.01000)
Supplement: Supplementary file 1 [file Data_Sheet_1.PDF]

Supplementary Material

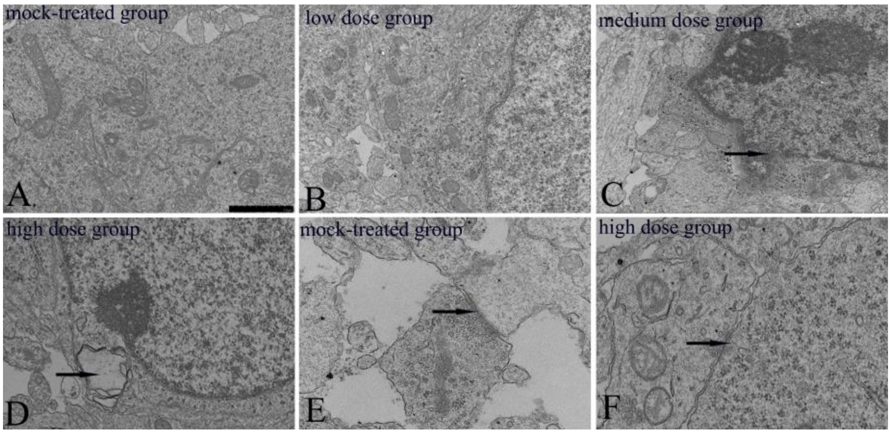

Supplementary Figure 1. Ultrastructural changes of cerebral cortex neurons and synapses in mice offspring after maternal exposure to $PM_{2.5}$ during pregnancy. (**A**) mock-treated group, normal neuron; (**B**) low-dosage group, no significant changes in the neuron; (**C**) The arrow shows the indistinct nuclear membrane; (**D**) the arrow shows the autophagic body; (**E**,**F**) the arrow shows the synapse. Bar = 1.0 µm.
